# Peripheral and Placental Prevalence of Sulfadoxine–Pyrimethamine Resistance Markers in *Plasmodium falciparum* among Pregnant Women in Southern Province, Rwanda

**DOI:** 10.4269/ajtmh.23-0225

**Published:** 2023-10-02

**Authors:** Muharib Alruwaili, Aline Uwimana, Reena Sethi, Monique Murindahabi, Emily Piercefield, Noella Umulisa, Andrew Abram, Erin Eckert, Kaendi Munguti, Aimable Mbituyumuremyi, Julie R. Gutman, David J. Sullivan

**Affiliations:** ^1^ Department of Clinical Laboratory Sciences, College of Applied Medical Sciences, Jouf University, Sakaka, Saudi Arabia;; ^2^ Department of Tropical Medicine, School of Public Health and Tropical Medicine, Tulane University, New Orleans, Louisiana;; ^3^ Department of Molecular Microbiology and Immunology, Johns Hopkins Bloomberg School of Public Health, Baltimore, Maryland;; ^4^ Malaria and Other Parasitic Diseases Division, Rwanda Biomedical Center, Kigali, Rwanda;; ^5^ Maternal and Child Survival Program/Jhpiego, Washington, District of Columbia;; ^6^ Roll Back Malaria, Ouagadougou, Burkina Faso;; ^7^ U.S. President’s Malaria Initiative, Malaria Branch, U.S. Centers for Disease Control and Prevention, Kigali, Rwanda;; ^8^ Maternal and Child Survival Program/Jhpiego, Kigali, Rwanda;; ^9^ U.S. Peace Corps, Kigali, Rwanda;; ^10^ RTI International, Washington, District of Columbia;; ^11^ U.S. President’s Malaria Initiative, U.S. Agency for International Development, Kigali, Rwanda;; ^12^ Malaria Branch, U.S. Centers for Disease Control and Prevention, Atlanta, Georgia

## Abstract

Intermittent preventive therapy during pregnancy (IPTp) with sulfadoxine–pyrimethamine (SP) is recommended in areas of moderate to high malaria transmission intensity. As a result of the increasing prevalence of SP resistance markers, IPTp-SP was withdrawn from Rwanda in 2008. Nonetheless, more recent findings suggest that SP may improve birthweight even in the face of parasite resistance, through alternative mechanisms that are independent of antimalarial effects. The prevalence of single nucleotide polymorphisms in *Plasmodium falciparum* dihydropteroate synthase (*pfdhps*) and dihydrofolate reductase (*pfdhfr*) genes associated with SP resistance among 148 pregnant women from 2016 to 2018 within Rwanda’s Southern Province (Huye and Kamonyi districts) was measured using a ligase detection reaction–fluorescent microsphere assay. The frequency of *pfdhps* K540E, A581G, and the quintuple (*pfdhfr* N51I + C59R + S108N/*pfdhps* A437G + K540E) and sextuple (*pfdhfr* N51I + C59R + S108N/p*fdhps* A437G + K540E + A581G) mutant genotypes was 90%, 38%, 75%, and 28%, respectively. No significant genotype difference was seen between the two districts, which are approximately 50 km apart. Observed agreements for matched peripheral to placental blood were reported and found to be 207 of 208 (99%) for *pfdhfr* and 239 of 260 (92%) for *pfdhps*. The peripheral blood sample did not miss any *pfdhfr* drug-resistant mutants or *pfdhps* except at the S436 loci. At this level of the sextuple mutant, the antimalarial efficacy of SP for preventing low birthweight is reduced, although overall SP still exerts a nonmalarial benefit during pregnancy. This study further reveals the need to intensify preventive measures to sustain malaria control in Rwanda to keep the overall incidence of malaria during pregnancy low.

## INTRODUCTION

An estimated 247 million clinical cases and 619,000 malaria-related deaths were reported worldwide in 2021.^1^ *Plasmodium falciparum* is the most prevalent and virulent of the human malaria species.^1^ Antimalarial drug resistance in *P. falciparum* parasites poses one of the greatest threats to malaria control. As a result of the widespread resistance to chloroquine (CQ) and sulfadoxine–pyrimethamine (SP), artemisinin-based combination therapies have been recommended for *P. falciparum* malaria treatment since 2006.^2^

Sulfadoxine–pyrimethamine plus amodiaquine (AQ) was used as a first-line antimalarial treatment of uncomplicated malaria in Rwanda from 2001 to 2006, at which time artemether–lumefantrine (AL) became the recommended first-line treatment.^2^ Resistance to SP is associated with polymorphisms in the *P. falciparum* dihydrofolate reductase (*pfdhfr*) and dihydropteroate synthase (*pfdhps*) genes.^3^^,^^4^ Antimalarial drug policy change may influence drug sensitivity as a result of the withdrawal of drug pressure on parasites. This was demonstrated in a 2003 study^5^ in Malawi where, 12 years after CQ withdrawal, no evidence of the *P. falciparum* CQ resistance transporter (*pfcrt* K76T) mutation was detected among isolates, compared with 85% reported in 1992, indicating that CQ efficacy may have been restored. In Rwanda, a slower recovery rate of CQ sensitivity has been reported after 14 years of cessation.^2^ However, despite 7 years of the presumed absence of SP pressure, a high level of SP resistance was reported in samples collected from Rwanda in 2015,^2^ which persisted in 2020.^6^ Sulfadoxine–pyrimethamine remains the only drug recommended by the WHO for intermittent preventive treatment during pregnancy (IPTp) to prevent the adverse consequences of malaria. Intermittent preventive treatment during pregnancy–SP is a recommended intervention in 35 sub-Saharan African countries.^1^^,^^7^

Although the quintuple mutant genotype, consisting of triple mutations of *pfdhfr* (N51I/S59R/S108N) and double mutations of *pfdhps* (A437G/K540E), is associated with clinical and parasitological SP treatment failure, IPTp-SP remains efficacious and confers some protection, particularly against low birthweight, even in areas with a high level of quintuple mutant genotypes,^8^ presumably because of the nonantimalarial effects of SP.^9^^,^^10^ However, IPTp-SP efficacy for preventing low birthweight is reduced when a high proportion of *P. falciparum* parasites acquire an additional *pfdhps* A581G mutation, leading to the sextuple mutant (*pfdhfr* N51I + C59R + S108N/p*fdhps* A437G + K540E + A581G).^8^^,^^11^ As a result of the documented high level of resistance, SP was withdrawn completely from all uses in Rwanda 2008, with cessation of the IPTp policy.^2^

Rwanda experienced a dramatic increase in malaria cases between 2012 and 2017, from 564,407 in 2012 to 4,746,985 in 2017—a more than an 8-fold increase.^12^ More than 79% of the malaria burden was reported from the eastern and southern provinces. Because it is not ethically appropriate to conduct an SP efficacy trial in regions with known high rates of treatment failure, resistance marker detection may be the only evidence to support the notion of reintroducing drugs after their withdrawal. We report SP resistance markers in *P. falciparum* isolates from pregnant women in a relatively high malaria transmission zone in Rwanda, collected during a cluster randomized controlled trial investigating intermittent malaria screening and treatment of positive patients during pregnancy, compared with routine antenatal care for reducing malaria prevalence at delivery. We also compared samples obtained from placental or peripheral blood to determine whether peripheral blood captured drug-resistant genotypes accurately.

## MATERIALS AND METHODS

### Study area and study participants.

**Figure 1.**
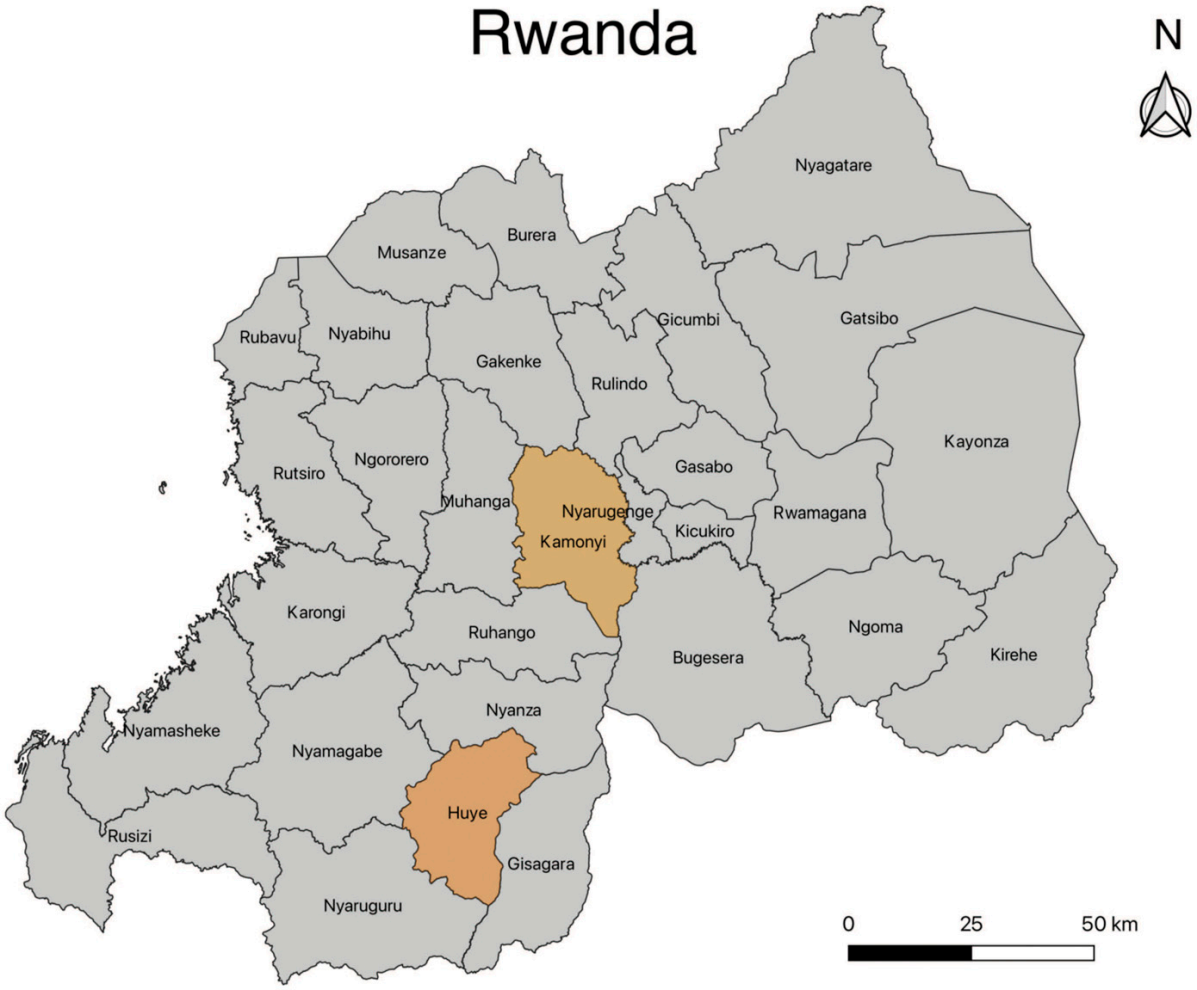
Map of Rwanda showing the study sites of the Huye and Kamonyi districts in Southern Province, Rwanda.

The study was conducted in two districts (Huye and Kamonyi), both of which are in the Southern Province of Rwanda (Figure 1). The Southern Province encompasses an area of 5,963 km^2^ with a population of about 2.6 million. Although malaria cases have declined in recent years, 1.8 million cases were reported in Rwanda in 2020 compared with 4.7 million cases in 2017. The Southern Province has the highest incidence in the country, with more than 450 cases per 1,000 people, compared with the national average of 198 per 1,000 people.^12^ In our study, we used clinical samples collected from pregnant women attending an antenatal care clinic in Rwanda between 2016 and 2018, and participating in a cluster-level randomized study to estimate the effectiveness of intermittent screening and treatment of malaria in pregnancy on maternal and birth outcomes (ClinicalTrials.gov identifier, NCT03508349).^13^ A total of 1,441 pregnant women participated in the study, which included rapid diagnostic testing locally. At the time of delivery, placental and peripheral blood samples for polymerase chain reaction (PCR) testing at later time were collected using a filter paper card.^13^ All available 1,267 placental or 1,347 peripheral samples were processed for PCR nucleic acid testing, with the 192 placental and 140 peripheral positive *P. falciparum* samples processed for further drug-resistant testing.

### Genotyping drug-resistant genes.

We genotyped the *P. falciparum* dihydrofolate reductase (*pfdhfr*) and dihydropteroate synthase (*pfdhps*) genes that confer resistance to pyrimethamine and sulfadoxine, respectively, using placental samples. Peripheral samples were compared with placental samples in matched pairs of PCR amplification from both sample locations. These genes were amplified by nested PCR and then a ligase detection reaction–fluorescent microsphere assay was performed to identify single nucleotide polymorphisms (SNPs) in the *pfdhfr* and *pfdhps* genes as described previously.^14^ We targeted four SNPs within the *pfdhfr* gene (N51I, C59R, S108N, and I164L) and five SNPs within the *pfdhps* gene (S436A, A437G, K540E, A581G, and A613S).

### Statistical analysis.

The prevalence of each SNP was calculated by dividing the number of samples carrying the SNP by the total number of samples genotyped successfully at that SNP. Each isolate was coded based on the presence or absence of a mutant allele, for which three categories were considered: wildtype, mutant, and mixed infection. In the final analysis, both mutant and mixed infection were coded as mutant alleles to generate the number of mutant alleles per codon. All statistical analyses were conducted using IBM SPSS Statistics 28.0 (SPSS Inc., Chicago, IL).

Kappa statistics were used to determine the degree of agreement between placental and peripheral matched samples for each locus using the formula

$$k=\frac{P_{0}-P_{e}}{1-P_{e}},$$

where *P*_0_ represents the overall proportion of observed agreement and *P_e_* represents the overall proportion of expected agreement.

## RESULTS

From the original study, placental samples were collected and analyzed successfully by reverse transcription–PCR from 1,267 women. The prevalence of placental infection at delivery by PCR from placental blood was 15% (192 of 1,267). whereas fingerstick peripheral prevalence was 10% (140 of 1,347). A total of 148 of 192 placental blood samples were genotyped successfully for *pfdhfr* and *pfdhps* genes, and 58 were matched to fully genotyped peripheral fingerstick blood.

### *pfdhps* gene.

The prevalence of mutant alleles (pure mutant and mixed) in placental samples at codons S436A, A437G, K540E, A581G, and A613S was 71%, 90%, 90%, 38%, and 3%, respectively (Table 1). The distribution of mutant alleles *pfdhps* S436A, A437G, K540E, A581G, and A613S were comparable across the two districts (Table 2).

**Table 1:** The prevalence of single nucleotide polymorphisms in the *pfdhps* and *pfdhfr* genes in Southern Province, Rwanda, (*N* = 148)

Gene/Haplotype	Huye (*n* = 47)			Kamonyi (*n* = 101)			Total, *n* (%)
	Wildtype, *n* (%)	Mutant, *n* (%)	Mixed, *n* (%)	Wildtype, *n* (%)	Mutant, *n* (%)	Mixed, *n* (%)	
*pfdhfr*							
51	2 (4)	44 (96)	0 (0)	2 (2)	97 (97)	0 (0)	141 (97)
59	5 (11)	39 (83)	3 (6)	6 (6)	93 (92)	2 (2)	137 (93)
108	1 (2)	46 (98)	0 (0)	0 (0)	101 (100)	0 (0)	147 (99)
164	43 (100)	0 (0)	0 (0)	88 (100)	0 (0)	0 (0)	0
*pfdhps*							
436	11 (26)	0 (0)	32 (74)	29 (31)	0 (0)	64 (68)	96 (71)
437	4 (9)	39 (91)	0 (4)	10 (11)	80 (86)	3 (3)	122 (90)
540	4 (9)	39 (91)	0 (4)	10 (11)	82 (87)	2 (2)	123 (90)
581	27 (69)	8 (21)	4 (10)	52 (59)	27 (31)	9 (10)	48 (38)

**Table 2:** Percentage of mutant (mixed and mutant) alleles

Gene/Haplotype	Codons	Huye, *n* (%)	Kamonyi, *n* (%)	Pearson’s χ^2^ test	*P* value
*pfdhfr*	51I	44 (96)	97 (98)	0.418	0.680
	59R	42 (89)	95 (94)	1.029	0.327
	108N	46 (98)	101 (100)	2.164	0.318
	164 L	ND	ND	–	–
	436A	32 (74)	64 (69)	0.313	0.576
*pfdhps*	437G	39 (91)	83 (89)	0.014	0.905
	540E	39 (91)	84 (89)	0.001	0.977
	581G	12 (31)	36 (41)	1.497	0.221
	613S	2 (5)	2 (2)	0.631	0.592
Triple *pfdhfr* (51I + 59R + 108N)	–	40 (85)	91 (90)	0.786	0.375
Double *pfdhps* (437G + 540E)	–	39 (83)	83 (82)	0.14	0.905
Quintuple mutant	–	35 (74)	76 (75)	0.10	0.919
Sextuple mutant	–	11 (23)	30 (30)	0.635	0.425
*pfdhfr*	51I	44 (96)	97 (98)	0.418	0.680

ND = not detected.

### *pfdhfr* gene.

The prevalence of mutant alleles (pure mutant and mixed) in placental samples for N51I, C59R, and S108N was 97%, 93%, and 99%, respectively (Table 1). All samples were found to carry the wildtype allele at *pfdhfr* I164L. The prevalence of the *pfdhfr* triple (51 + 59 + 108) haplotype was 89% in the Huye District and 90% in the Kamonyi District; however, the distribution of haplotype combinations was comparable across the two sites (Table 2).

### Haplotype combinations.

The prevalence of quintuple (*pfdhfr* 51 + 59 + 108/*pfdhps* 437 + 540) and sextuple (*pfdhfr* 51 + 59 + 108/*pfdhps* 437 + 540 + 581) mutant genotypes in placental samples was 75% and 28%, respectively. The distribution of mutant alleles and haplotype combinations were comparable across both Huye and Kamonyi districts (Table 2). Among monoclonal infections, the prevalence of quintuple mutants and sextuple mutants was 78 of 113 (69%) and 29 of 113 (26%), respectively, whereas for polyclonal infections, the quintuple and sextuple mutant prevalence was 27 of 35 (77%) and 11 of 35 (31%), respectively.

### Concordance of placental and peripheral blood samples for both *pfdhfr* and *pfdhps* drug-resistant genotypes.

Matched peripheral-to-placental blood detection of drug-resistant mutants were genotyped for each locus in the *pfdhfr* and *pfdhps* genes. The observed agreements for the four loci in *pfdhfr* (51 + 59 + 108 + 164) were 207 of 208 (99%), and for the five loci in *pfdhps* (436 + 437 + 540 + 581 + 613) were 239 of 260 (92%) (Table 3). The peripheral blood sample detected all *pfdhfr* and all *pfdhps* drug-resistant mutants correctly except at the S436 loci. Four SNPs (*pfdhfr* N51I, C59R, and I164L, and *pfdhps* A613S) exhibited perfect agreement. The kappa values for *pfdhfr* S108N and *pfdhps* S436, A437G, K540E, and A581G were 0.60, 0.38, 0.91, 0.81 and 0.80, respectively. The overall degree of agreement between placental and peripheral blood in classifying SNPs associated with sulfadoxine and pyrimethamine was strong (κ = 0.83).

**Table 3:** Placental and peripheral blood agreement on drug resistance genotyping

Polymorphism	Capillary	Placental, *n*				Observed agreement, *n*/*N* (%)	Kappa value
		Mutant	Wildtype	Mixed	Total		
*pfdhfr* 51	Mutant	50	0	0	50	–	–
	Wildtype	0	1	0	1	–	–
	Mixed	0	0	0	0	–	–
	Total	50	1	0	51	51/51(100)	1
*pfdhfr* 59	Mutant	49	0	0	49	–	–
	Wildtype	0	5	0	5	–	–
	Mixed	0	0	1	1	–	–
	Total	49	5	1	55	55/55 (100)	1
*pfdhfr* 108	Mutant	53	0	0	53	–	–
	Wildtype	0	1	0	1	–	–
	Mixed	1	0	0	1	–	–
	Total	54	1	0	55	54/55 (98)	0.66
*pfdhfr* 164	Mutant	0	0	0	0	–	–
	Wildtype	0	47	0	47	–	–
	Mixed	0	0	0	0	–	–
	Total	0	47	0	47	47/47 (100)	1
*pfdps* 436	Mutant	0	0	0	0	–	–
	Wildtype	0	8	7	15	–	–
	Mixed	0	6	31	37	–	–
	Total	0	14	38	52	39/52 (75)	0.32
*pfdps* 437	Mutant	45	1	0	46	–	–
	Wildtype	0	6	0	6	–	–
	Mixed	0	0	0	0	–	–
	Total	45	7	0	52	51/52 (98)	0.91
*pfdps* 540	Mutant	47	1	0	48	–	–
	Wildtype	0	5	0	5	–	–
	Mixed	0	1	0	1	–	–
	Total	47	7	0	54	52/54 (96)	0.81
*pfdps* 581	Mutant	11	1	0	12	–	–
	Wildtype	0	35	1	36	–	–
	Mixed	1	2	2	5	–	–
	Total	12	38	3	53	48/53 (91)	0.75
*pfdps* 613	Mutant	0	0	0	0	–	–
	Wildtype	0	49	0	49	–	–
	Mixed	0	0	0	0	–	–
	Total	0	49	0	49	49/49 (100)	1

## DISCUSSION

Despite transitioning from SP + AQ to AL as the first-line treatment in 2006, and discontinuing IPTp-SP in 2008, there continued to be a relatively high proportion of both quintuple (75%) and sextuple (28%) mutants in samples collected in southern Rwanda from 2016 to 2018, despite the limitation of collection 5 to 7 years ago.^2^ Overall, the prevalence of *pfdhps* A581G was 38%. Data from neighboring Uganda highlight the focal nature of the A581G mutant, as prevalence varied from 5% to 46% in 2018 and 2019, depending on the province.^15^ Although the prevalence of the quintuple mutant in our study, conducted in 2016 to 2018, is similar to that reported from Rwanda’s Ruhuha (Eastern Province) and Mubuga (Western Province) in 2015 (74%), the prevalence of the sextuple mutant genotype in our sample was greater (28% compared with 18%), with a corresponding greater prevalence of *pfdhps* A581G (from 24% in the 2015 study to 38% in our sample).^2^ Because resistance markers may be highly focal, the geographic difference in study sites may account for the difference in the prevalence between our findings and the previous study from Rwanda.^2^ Thus, it is not possible to state that this is definitive evidence of increasing resistance over time. However, older data from the Democratic Republic of the Congo, which shares a border with Rwanda, demonstrated an increase in *pfdhps* K540E/A581G (not defined as a sextuple mutant) from 2% in 2007 to 18% in 2013, suggesting that the increase in Rwanda may be real.^16^ The sextuple mutant has been associated with increased treatment failure, as well as delivery of low-birthweight infants among women taking IPTp with SP.^17^^,^^18^ A recent, large clinical trial with nearly 1,500 participants in each arm compared SP to dihydroartemisinin–piperaquine alone or combined with a single dose of azithromycin in settings with 20% to 30% prevalence of the sextuple mutant. Despite the relatively high prevalence of the sextuple mutant, adverse pregnancy outcomes were reported more frequently in the groups receiving dihydroartemisinin–piperaquine.^10^

Recovery of wildtype (susceptible) alleles after withdrawal of antimalarial drugs has been reported in several countries, although timelines have varied.^2^^,^^5^ For instance, high rates of recovery of CQ susceptibility have been reported in Malawi, where parasites reverted to wildtype at the *pfcrt* K76T codon responsible for CQ resistance 12 years after cessation of CQ use.^5^ In Rwanda, a slower recovery rate has been reported, with the prevalence of mutant *pfcrt* K76T declining from 74% in 2010 to 49% in 2015, suggesting an approximate 25% recovery of wildtype *pfcrt* K76T after a decade of CQ withdrawal.^2^^,^^19^ For SP, we observed a high level (> 92%) of *pfdhfr* N51I, C59R, and S108N and more than 89% of *pfdhps* A437G and K540E, which is similar or greater than reported previously in Rwanda.^2^ This demonstrates the persistence of high-level SP resistance even years after SP withdrawal, similar to what has been reported in Uganda and Tanzania, although a decrease in *pfdhfr* and *pfdhps* mutant alleles were reported in Ethiopia after SP withdrawal.^20^^–^^22^ The persistence of high levels of SP resistance in Rwanda even in the absence of SP pressure for almost a decade may be a result of gene flow from neighboring countries or the continued use of SP or other antifolate inhibitors such trimethoprim and sulfamethoxazole, which may have cross-resistance with SP.^23^ Alternatively, the SP mutations may not have the same fitness cost as those for CQ, and there may thus be less pressure to revert.

We found a high level of agreement between placental and peripheral blood SP drug-resistant mutants. Importantly, testing of peripheral blood did not miss mutant SP *P. falciparum* parasites for *pfdhfr* and all but one of the *pfdhps* genes, suggesting that maternal peripheral blood would be sufficient and provide a good measurement for the prevalence of SP resistance markers in pregnant women. A small study in Gabon^24^ saw greater than 90% agreement in SP drug-resistant mutations from 22 matched samples collected in 2005 and 2006. A similar study in Ghana^25^ with 294 matched pairs collected in 2000 and 2001 indicated 83% agreement between peripheral and placental blood samples that were influenced by parasite density, number of mutations, and fever. The agreement increases inversely with the number of alleles, such that the multiallelic MSP2 indicated less agreement between placental and peripheral samples.^26^

## CONCLUSION

Our study demonstrated a high level of focal SP resistance, with a 23% and 30% prevalence of the sextuple mutant in the Huye and Kamonyi districts, respectively, despite nearly a decade of a presumed absence of SP pressure. At this level of resistance, the efficacy of SP for prevention of low birthweight is likely compromised. As drug resistance can be quite focal, resistance testing would be indicated prior to implementing any SP-containing preventive strategy in Rwanda.
